# Characterization of chemokine and cytokine expression pattern in tuberculous lymphadenitis patient

**DOI:** 10.3389/fimmu.2022.983269

**Published:** 2022-11-10

**Authors:** Bernadette Dian Novita, Yudy Tjahjono, Sumi Wijaya, Imelda Theodora, Ferdinand Erwin, Stefan Wilson Halim, Bobby Hendrawan, David Karunia Jaya, Paul L. Tahalele

**Affiliations:** ^1^ Faculty of Medicine, Widya Mandala Surabaya Catholic University, Surabaya, Indonesia; ^2^ Faculty of Pharmacy, Widya Mandala Surabaya Catholic University, Surabaya, Indonesia

**Keywords:** C-C chemokine receptor and ligands, interleukins, Stat 3, SOCS 3, lymphadenitis tuberculosis

## Abstract

**Introduction:**

C-C chemokine receptor-2 (CCR-2) and C-C chemokine ligand-5 (CCL-5) play an important role in the migration of monocytes, macrophages, dendritic cells, and activated T cells against *Mycobacterium tuberculosis* (M.tb). Meanwhile, signal transducer and activator of transcription 3 (STAT-3) and suppressor of cytokine signaling 3 (SOCS-3), activated by interleukin (IL)-6 and IL-10 in tuberculosis (TB) infection, play an important role in phagocytosis, inflammation, and granulomatous-forming processes that may lead to TB treatment success or failure. However, there are no data about the expression of those markers in tuberculous lymphadenitis. The characterization of those markers is very critical to put a fundamental basis to understand the homing mechanism of tuberculous lymphadenitis.

**Aim of study:**

The specific objective of this study is to characterize the expression pattern of CCR-2-CCL-5, IL-6, IL-10, STAT-3, and SOCS-3 in tuberculous lymphadenitis.

**Methods:**

The study was performed on 27 cases of tuberculous lymphadenitis node biopsies. The diagnosis of tuberculous lymphadenitis was based on the clinical criteria and the presence of the histological feature characteristic of TB granulomas. Afterward, immunohistochemistry was stained with CCR-2, CCL-5, IL-6, IL-10, STAT-3, and SOCS-3. A semiquantitative analysis of IHC images was performed to examine protein expression in stained preparations. The expression was also manually counted.

**Results:**

Compared with the normal area, both lymphocytes and macrophages expressed strongly CCR-2-, CCL-5, and IL-6, while IL-10, STAT-3-, and SOCS-3- were expressed lowly. There was a strong positive correlation between CCR-2 with IL-6 (p = 0,83) and IL-10 (p = 0,83).

**Conclusion:**

The chronic infection process of tuberculous lymphadenitis was characterized by the expression of IL-10^low^, STAT-3^low^, SOCS-3^low^, CCR-2^high^, CCL-5^high^, and IL-6^high^.

**Clinical Trial Registration:**

Clinicaltrials.gov, identifier NCT05202548.

## Background

Tuberculosis (TB) is a chronic infection caused by *Mycobacterium tuberculosis* (M.tb). TB remains one of the “global health emergency” diseases (1). Nowadays, the evidence of TB new cases is increasing. Previous studies show that this situation was associated with the rising number of patients with immunocompromised conditions such as HIV, diabetes mellitus (DM), cancer, and autoimmune diseases (2, 3). Most commonly, TB affects the lung. However, TB can also affect other organs, a form known as extrapulmonary TB (EPTB), which accounts for approximately 5% of TB incidence. It affects lymph nodes (LNs), pleura, bone and joints, the gastrointestinal tract, the urogenital tract, meninges, and other parts of the human body (4, 5). Tuberculous lymphadenitis is the most common form of EPTB.

The histopathological features of the LNs with tuberculous lymphadenitis are composed of caseous necrosis, granulomas, epithelioid cells, lymphocytes, and histocytes. The characteristic of those specific histological features is confirmed with the acid-fast bacilli (AFB) direct smear result. Epithelioid cells may also fuse to form multinucleated giant cells, and central necrosis is evident in necrotizing granulomas. Caseous necrosis and granulomatous formation are two specific pathological features of LN TB (6, 7).

In pulmonary TB, macrophages play important roles in the recognition of pathogen-associated molecular patterns (PAMPs) present at the M.tb surface by various pattern recognition receptors (PRRs) in the surface of macrophages, such as C-type lectin receptors (CLR/CTL), Fc receptors (FcRs), and Toll-like receptors (TLRs) (8). This response induces IFNγ and TNFα to activate macrophages [classified activated macrophage (CAM)] (9) against M.tb. Macrophages regulate cytokines, such as IL-10 and IL-6, that are related to STAT-3 and SOCS-3 activation and inhibition. Meanwhile, in tuberculous lymphadenitis, some studies show that it relates to increase (TNFα and IL-17A) and decrease (IL-1α, IL-1β, and IL-18) (10, 11). However, the immune response in pulmonary TB and tuberculous lymphadenitis is different and poorly understood due to the lymphocyte function that also takes an important role as macrophages.

The interaction between M.tb and the migrating cells changes the chemokine receptor expression that guides cells at the inflammatory foci. This makes granuloma formation or the travel to the regional LN, where the antigen presentation and adaptive immune response development occur. This process is crucial in developing a host response to pathogen infection (M.tb) (12–15). Regulating M.tb infection depends on the interaction between T lymphocytes and infected macrophages. In M.tb infection, T cells are recruited to the infection site predominantly by the modulation of chemokine receptors (CRs) such as C-C chemokine receptor-2 (CCR-2) and C-C chemokine receptor-5 (CCR-5).

In TB infection, CCR-2 plays an essential role in attracting macrophages to the infected site (16). CCR-2 is a chemokine receptor, which is normally expressed on monocytes, macrophages, dendritic cells (DCs), and activated T cells. During TB infection, the activation of CCR-2 with chemotaxis proteins such as C-C chemokine ligands CCL-5, CCL-7, and CCL-12 could induce chemotaxis targeted at M.tb-infected cells such as local epithelial, mesothelial, and inflammatory cells (14, 17, 18). Low CCR-2 expression in the lymphocytes of TB patients results in delayed granuloma formation and incomplete granuloma formation (16). A meta-analysis found that the CCR-2 gene improves protection against mycobacterium infection (19). CCR-2 in alveolar macrophages is important in attracting macrophages to form granuloma in TB infection. The absence of CCR-2 expression in the alveolar macrophages of CCR2^-/-^ mice leads to failure in granuloma formation and increased necrotizing lesions in TB infection (20). Low CCL-5 levels in TB infection cause the dysfunction of various immune cells, which is the primary factor for M.tb bacteria resistance (21). CCL-5 is an important chemokine for Th1 migration; however, it does not affect Th2 migration (22). Aside from CCR-2 and CCL-5, transcription factor STAT-3 (signal transducer and activator of transcription-3) and its product SOCS-3 (suppressor of cytokine signaling-3) play essential roles in the pathogenesis of TB infection. In the early stages of TB infection, STAT-3 is rapidly activated by IL-10. However, STAT-3 could be activated by other cytokines such as IL-6, IL-21, IL-23, IL-27, and Granulocyte colony stimulating factor (G-CSF), which modulate the cellular response of macrophages and DCs in TB infection (23). STAT-3 has also been found to inhibit the production of inflammatory cytokines such as IL-6, TNF-α, IFN-γ, and nitric oxide (NO) (24). The action of STAT-3 has a disadvantaged outcome on M.tb-infection therapy.

During Tb infection, SOCS-3 expression in macrophages is elevated after the stimulation of TLR-2 with the mycobacterium cell wall components, such as a-crystallin-related protein 1(ACR1), phosphatidyl-myo-inositol mannosides (PIM2), and protein–proline–proline–glutamic acid-18 (PPE-18) (25–27). SOCS-3 provides T cells a protection effect from M.tb. Mice with a lack of SOCS-3 on myeloid cells (*SOCS-3^fl/fl^ lysM cre*) was more susceptible to M.tb infection. In the absence of SOCS-3, IL-6 inhibits the production of IL-12 in Antigen Presenting Cells (APC), decreasing the Th1 differentiation and impairing the elimination of M.tb (28).

Taken together, CCR-2, CCL-5, IL-6, IL-10, STAT-3, and SOCS-3 are very critical in M.tb immunopathogenesis. However, it is unknown whether those pathways could be effective against tuberculous lymphadenitis. Furthermore, there was a lack of information about the immunology process of extrapulmonary TB, especially tuberculous lymphadenitis. Since tuberculous lymphadenitis was difficult to diagnose, treat, and monitor the therapy result, the specific objective of this study is to explain the expression pattern of CCR-2-CCL-5, IL-6, IL-10, STAT-3, and SOCS-3 in tuberculous lymphadenitis patients. The clinical approach based on immunological parameters could be explicitly implemented for tuberculous lymphadenitis patients by expression pattern analysis.

## Methods

The study was performed on 27 cases of tuberculous lymphadenitis biopsies. The diagnosis of tuberculous lymphadenitis was based on 1) the clinical criteria (7, 29) and 2) the presence of the histological feature characteristic of tuberculous granulomas. LN intumescence was one of the clinical criteria in this study. Open biopsy was done to establish the specific causative of it. A direct smear for AFB had also been done during the operation to confirm tuberculous lymphadenitis. Those cases were obtained from the archives of the Department of Pathology, Vincentius A Paulo Surabaya Hospital, Indonesia. Ethical clearance was obtained from the Widya Mandala Surabaya’s Health Research Ethics Committee Ref no: 143/WM12/KEPK/DOSEN/T/2021. This study was also registered in Clinicaltrials.gov with ID no. NCT05202548.

### Immunohistochemical assay for tuberculous lymphadenitis specimen

Each specimen was cut into 5-µm-thick parallel sections. Two sections were stained. The first section was stained with hematoxylin and eosin. At the same time, the second section was stained with the Ziehl–Nielsen stain for AFB. After deparaffinization and rehydration, the sections were briefly microwaved for antigen retrieval using a citrate buffer, pH 6·0, at 750 W for 10 min, and at 350 W for 15 min. After being cooled down for 20 min at room temperature, the sections were incubated with hydrogen peroxide for 10–15 min and then washed two times with the buffer. Then, the sections were incubated separately with primary antibodies according to the manufacturer’s protocol. The antihuman antibodies (anti-CCL-5 polyclonal antibody Catalog No. E-AB-17864; anti-CCR-2 polyclonal antibody Catalog No. E-AB-40273; anti-IL-6 polyclonal antibody Catalog No. E-AB-30095; anti-IL-10 polyclonal antibody Catalog No. E-AB-40016; anti-STAT-3 monoclonal antibody Catalog No. E-AB-22119; and anti-SOCS-3 polyclonal antibody Catalog No. E-AB-65703) were purchased from Elabscience. Then, it was followed by a 10-min incubation with biotinylated goat antipolyvalent and 10 min with streptavidin peroxide at room temperature. The last step was to apply a mixture of 40 μl of DAB Plus Chromogen and 2 ml of a DAB Plus substrate (Elabscience, Houston, TX, USA) into tissue for 15 min. The cells expressed CCL-5, CCR-3, IL-6, IL-10, STAT-3, SOCS-3 were manually counted in 10 fields of view, using ×400 magnification by two pathologists.

### Relative expression measurement of tuberculous lymphadenitis specimen

A semiquantitative analysis of immunohistochemistry (IHC) images was performed with ImageJ software to examine protein expression in stained preparations, following the protocols developed by Crowe and Yue (30). In brief, the resulting image was saved as a.jpg file. Using ImageJ software, each file was analyzed using deconvolution methods. The threshold value was determined using five graphs to reduce the background and unspecific signal. According to our data, the threshold value is 155. After adjusting each data threshold, the data value was measured by documenting the “mean grey value,” which represents the quantified specific signal. GraphPad software was used to present the collected data, including the mean and standard deviation for each data set. The “non-expressed” region of each patient’s preparation was analyzed and presented as a “negative-control” in the diagram box of each parameter to validate further the specific expression of macrophages and lymphocytes in granuloma (30).

### Correlation analysis

Statistical analysis was generated with Graphpad Prism and performed using the non-parametic Kolmogorov–Smirnov test. To examine the correlation between each variable, SPSS 22 software was used. Data were presented in the mean ± standard deviation. The data were analyzed using Student’s t-test. Levene’s variance equivalence test was used to assess the assumption of the homogeneity of variance. The Spearman rank correlation coefficient was used to analyze the correlation between CCR2 expression with IL6 or with IL10. Statistical analysis is two sided, and p-values less than 0.05 are considered statistically significant.

## Results

### CCR-2, CCL-5, IL-6, IL-10, STAT-3, and SOCS-3 are expressed in the granuloma of a tuberculous lymphadenitis patient

**Figure 1:**
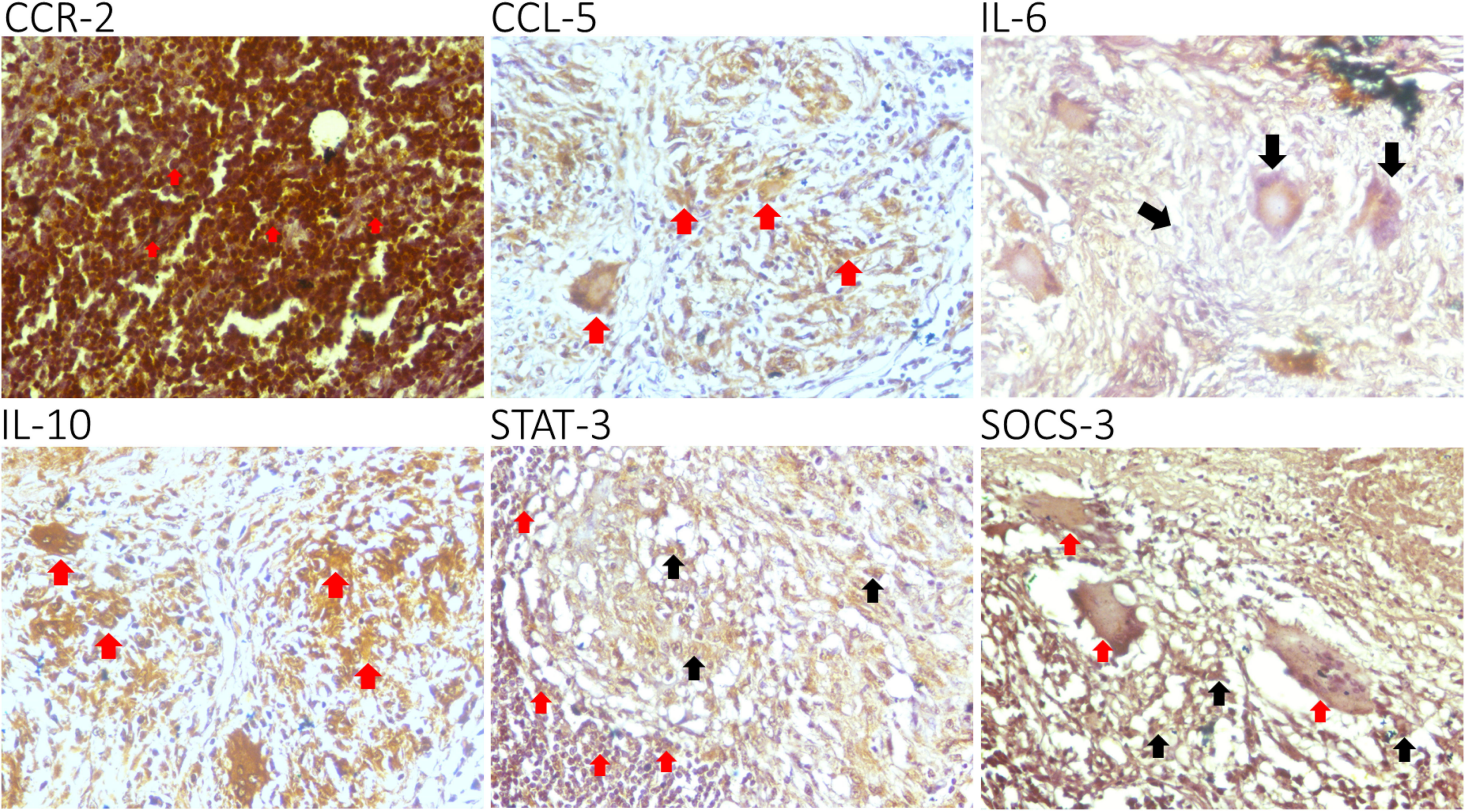
Epression of C-C chemokine receptor-2 (CCR-2), C-C chemokine ligand-5 (CCL-5), interleukin (IL)-6, IL-10, STAT-3, and SOCS-3 in lymphocytes (red arrow). A representative biopsy specimen of tuberculous lymphadenitis patient: CCR-2, CCL-5, IL-6, IL-10, STAT-3, and SOCS-3 are expressed in the cytoplasmic areaa. CCR-2 and IL-6 are strongly expressed in the tubercle, while the other markers, especially STAT-3 and SOCS-3, are expressed weakly. GIant cells (group of mature lymphocytes) could also express STATS-3 and SOCS-3 (black arrow).

In every biopsy specimen of 35 tuberculous lymphadenitis patients, CCR-2, CCL-5, IL-6, IL-10, STAT-3, and SOCS-3 were expressed in the cytoplasmic area. CCR-2 and IL-6 were strongly expressed in the granuloma, while the other markers, especially STAT-3 and SOCS-3, were expressed weakly (**Figure 1**)

**Figure 2:**
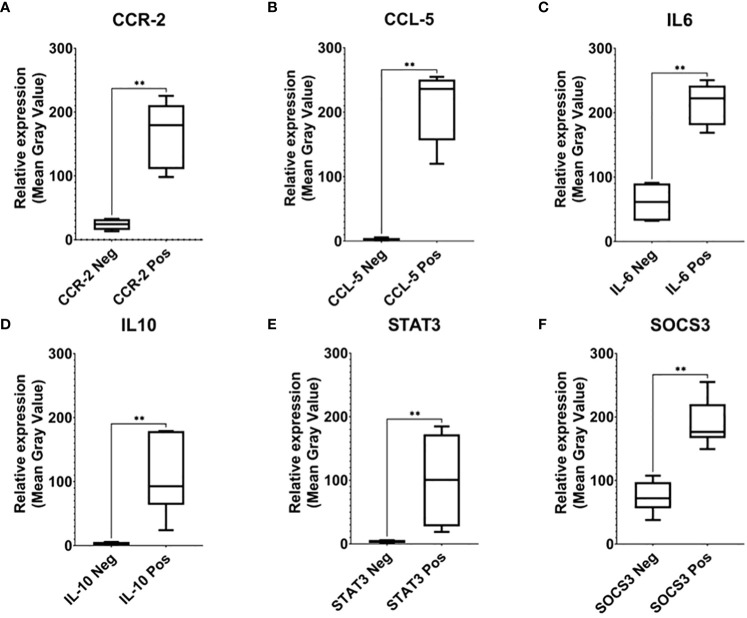
Relative expression analysis of CCR-2 **(A)**, CCR-5 **(B)**, IL-6 **(C)**, IL-10 **(D)**, STAT3 **(E)**, and SOCS-3 **(F)**. To confirm the specificity of the expression pattern, we further compared the strongly expressed area with the uninfected area in the same biopsy tissue (neg). The expression pattern of those markers was significantly higher than the negative area, which indicates signal specificity (** indicated 0.001 ≤ P < 0.01).

To confirm the specificity of the expression pattern, we further compared the strongly expressed area with the uninfected area in the same biopsy tissue (**Figure 2**). The expression pattern of those markers was significantly higher (P<0,01) than the negative area.

### The same number of lymphocyte and macrophage populations expressed CCR-2-, CCL-5, IL-6, and IL-10, while STAT-3- and SOCS-3-positive macrophages were slightly lower than lymphocytes

**Figure 3:**
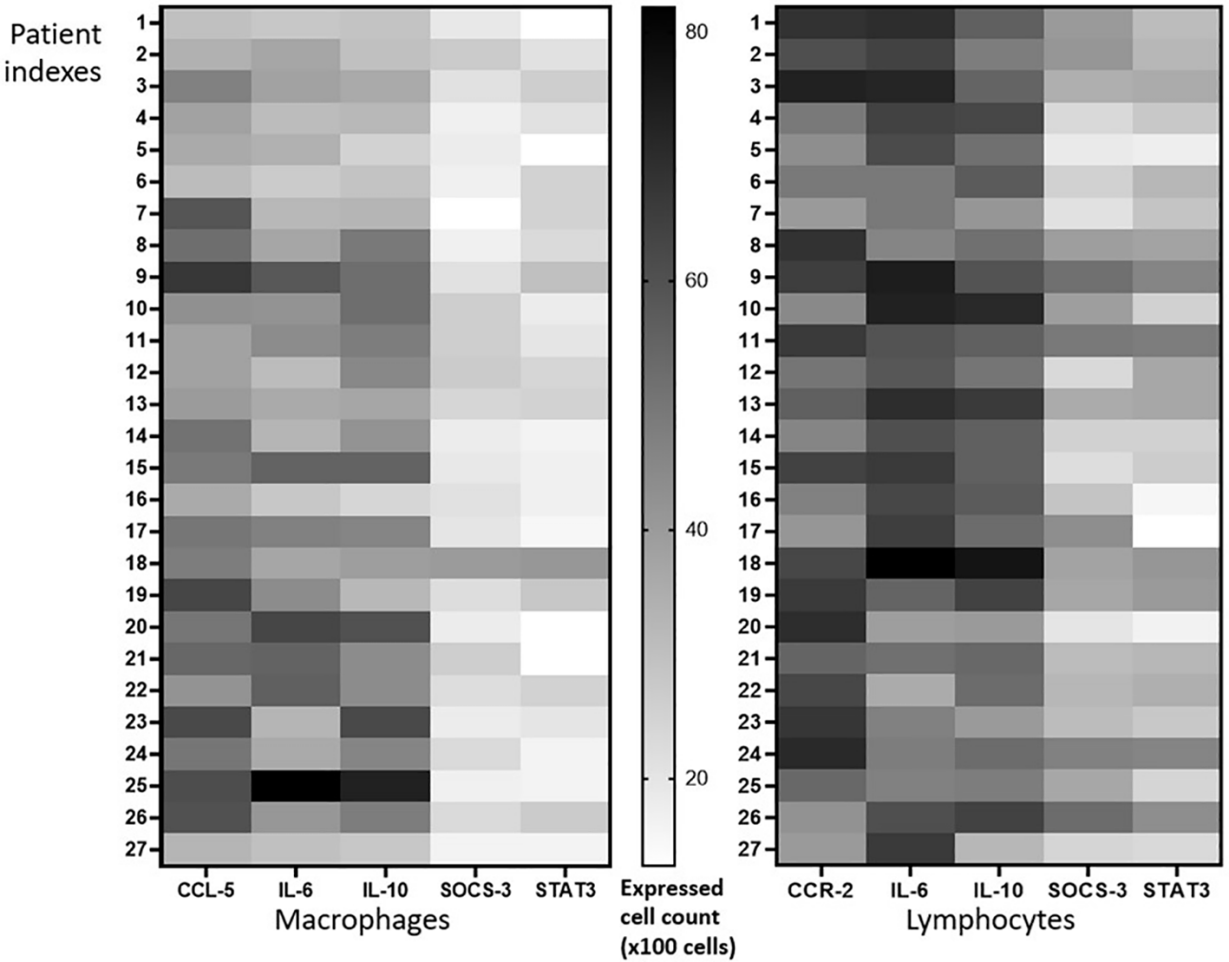
Expression pattern analysis. The same number of populations of lymphocytes and macrophages expressed CCR-2-, CCL-5, IL-6, and IL-10, while STAT-3- and SOCS-3-positive macrophages were slightly lower than lymphocytes.

**Figure 4:**
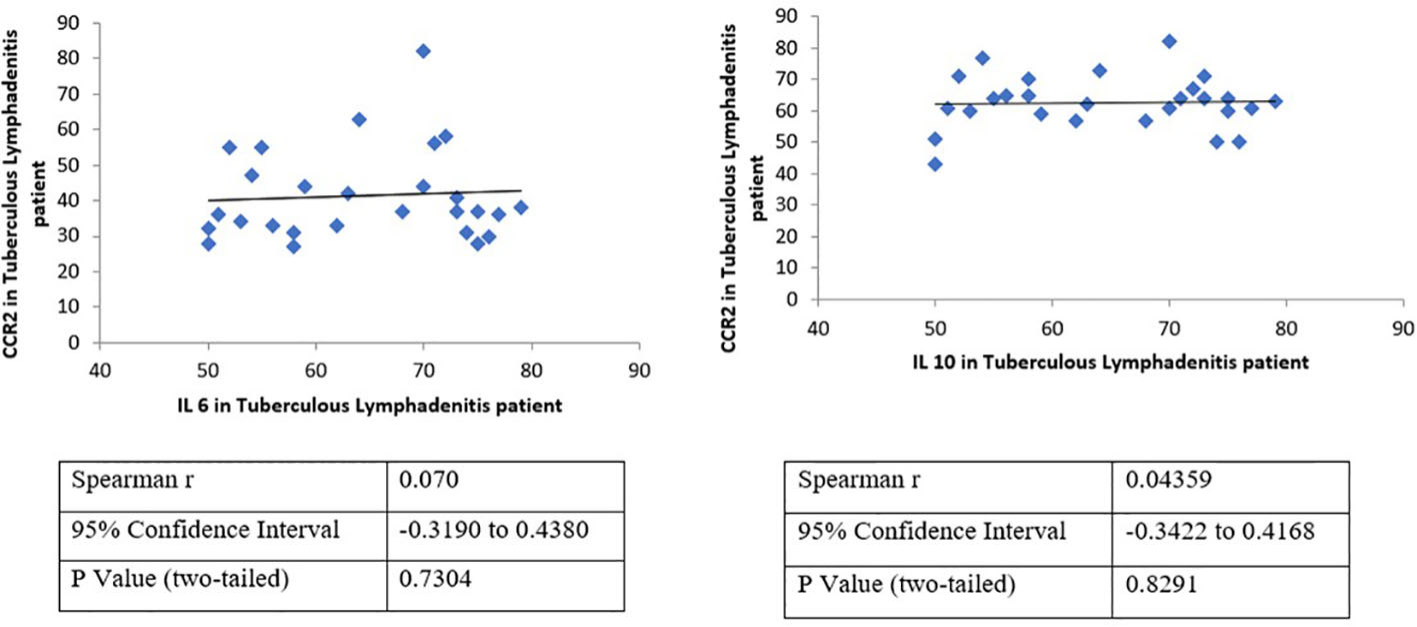
Correlation analysis validates the association between the expression of CCR2 and IL-6 or IL-10 in tuberculous lymphadenitis patients. A positive correlation between CCR2–IL-6 and CCR2–IL-10, which was never reported before for tuberculous lymphadenitis patients, has been demonstrated. However, the correlation of CCR-2 and CCL-5 was statistically insignificant. CCR-2 has strong statistical correlation to IL-6 (p = 0,73) and IL-10 (p = 0,83).

To compare whether the lymphocyte or macrophage population expressed the marker proteins (CCR-2-, CCL-5, IL-6, IL-10, STAT-3- and SOCS-3) predominantly, we manually counted the lymphocytes and macrophages that have a strong marker protein expression. The heatmap graphic (**Figure 3**) shows the population density of both lymphocytes and macrophages in each patient. The total cell population expressing the marker proteins is analyzed in **Figure 4**.

Meanwhile, many CCR-2-positive lymphocyte and CCL-5-positive macrophages populations were observed in the caseous area; both lymphocytes or macrophages predominantly express IL-6 and IL-10. It is also shown clearly that there are a small number of SOCS-3- and STAT-3-positive lymphocytes and macrophages. Overall, both lymphocytes and macrophages express the same number of markers of protein-positive cells. Although insignificant, macrophages tend to have a smaller number of SOCS-3- and STAT-3-positive cell populations than lymphocytes (**Figure 3**). Interestingly, further analysis showed a positive correlation between CCR2–IL-6 and CCR2–IL-10 (**Figure 4**), which was never reported before for tuberculous lymphadenitis patients. However, the correlation between CCR-2 and CCL-5 was statistically insignificant. CCR-2 had strong statistical correlation to IL-6 (p = 0,73) and IL-10 (p = 0,83), as shown in **Figure 4**.

## Discussion

The expression of CCR-2 is relevant with the recruitment of immune cells (phagocytic cells) to the M.tb site of invasion. It is well known that macrophages and lymphocytes are the most predominant cell population that resides in the lung TB granulomatosis (29, 31). To understand further the pathological mechanism of tuberculous lymphadenitis, the preliminary assessment of macrophages and lymphocytes is very critical. Interestingly, our results observed the accumulation of those cells in the tuberculous lymphadenitis biopsies, which is theoretically similar with the classical lung TB mechanism.

In this study, CCR-2 and CCL-5 were highly expressed in tuberculous lymphadenitis samples. However, the correlation between CCR-2 and CCL-5 was statistically insignificant. This reflects the relatively long infection process of M.tb in the host and the ability of macrophages in isolating TB infection only in the LN area (**Figures 1A–D**). IL-6 was also highly expressed, and the high expression of IL-6 was related to CCL5. Interestingly, we observed that IL-10, STAT-3, and SOCS-3 were expressed weakly in the samples. Although there was no statistically significant association between CCR-2 and CCL-5 on STAT-3 and SOCS-3 activation, IL-6 had the highest correlation to STAT-3. The low expression of SOCS-3 was not correlated with the low expression of STAT-3 nor the high expression of chemokine receptor–ligand and IL-6. The low expression in IL-10, STAT-3, and SOCS-3 expression may lead to tuberculous lymphadenitis (**Figures 2**, **3**). In pulmonary TB, the high concentrations of IL-6/IL-10 and STAT-3 led to the impaired function of T cells. Meanwhile, the chronic inflammatory process was found in the LN tissue, causing a very high expression of chemokine receptors and ligands, IL-6 expression. However, the expression of IL-10, STAT-3, and SOCS-3 was low, and those phenomena were interesting and assumed due to continuous TH17 impairment since the TB infection process occurred.

The high expression of CCR-2 and CCL-5 related to the complex interaction in response to M.tb infection was followed by the immunological process, such as macrophage activation, which led to a phagolysosome process. This process secretes cytokines like the tumor necrosis factor (TNF), interferon alpha (IFN-α), IFN-β, IL-6, IL-12, IL-1β, and the production of antimicrobial reactive nitrogen intermediates (ROIs), which aim to eliminate the M.tb bacteria. STAT-3 phosphorylation was then activated by IL-6. CCR-2 and CCR3 are expressed on macrophages or monocytes, DCs, and T lymphocytes, and they induce their migration and localize the site of inflammation. The migration of macrophages to the site of infection is important for the initial immune response against M.tb infection. Macrophages phagocytize the bacteria and provide important cellular information and a source of antigenic stimulation. Afterward, macrophages enhance the production of inflammatory chemokines that recruit more cells to make granuloma and modulate the migration and localization of cells within peripheral LNs (32).

**Figure 5:**
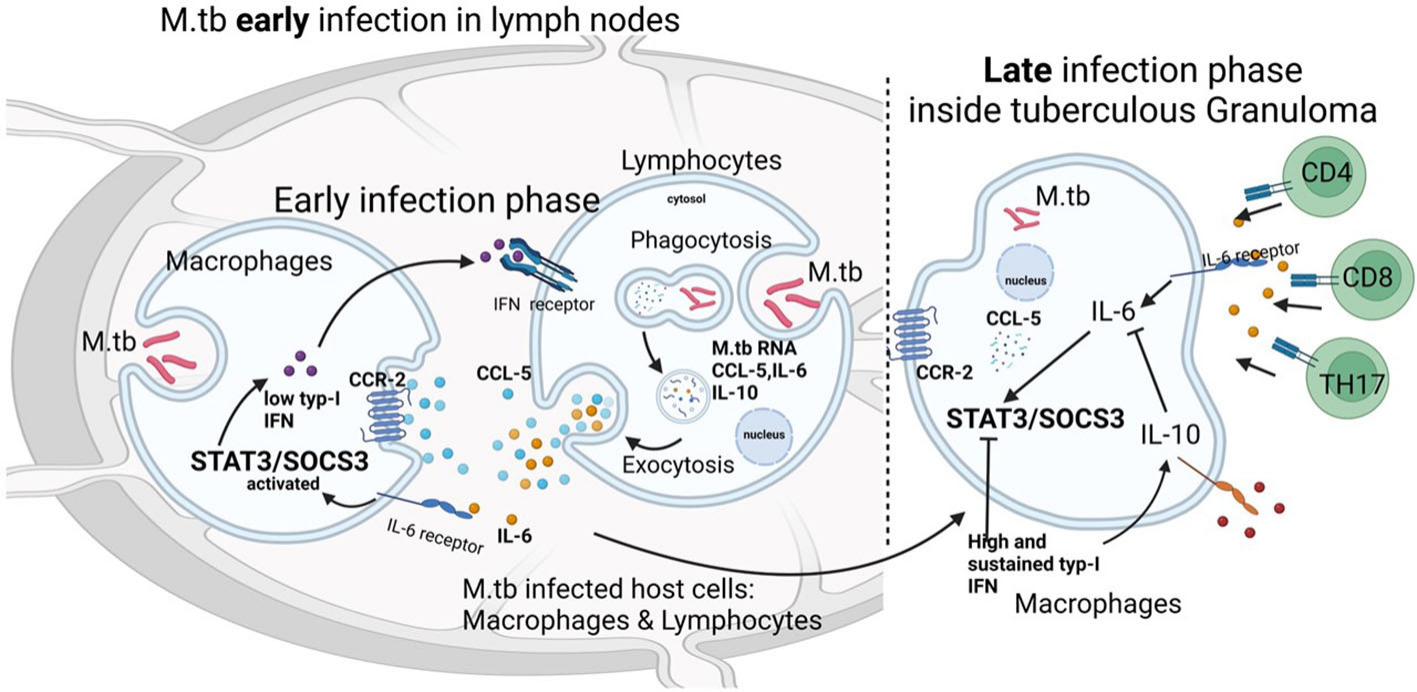
Relationship between the chemokine–ligand receptors IL-6 and IL-10 with STAT3 and SOCS3 in tuberculous lymphadenitis. In the early stages of infection, an increase in IL-6/IL-10 can also increase STAT-3 and SOCS-3, its regulator. This activation of STAT-3/SOCS-3 is related to an immunological response to TB infection. Chronic inflammation after TB infection reduces the function of T cells, especially T_H_17 cells, and this explains the low STAT-3/SOCS-3 expression in this study. Another theory that may also explain that low STAT-3/SOCS-3 expression is due to high and sustained type I interferon (IFN) production in the late phase of TB infection. High IFN inhibits the activation of STAT-3 by IL-6.

The low SOCS-3 expression observed in this manuscript is a phenotypical manifestation of the IL-6/STAT-3-activation dysregulation, which leads to tuberculous lymphadenitis. This situation might be triggered by the M.tb chronic infection process since SOCS-3 expression is essential for resistance against tuberculous infection (28). In the previous study, STAT-3 expression was high (33). Meanwhile, the expression was low in this study. IL-6 is one of the important activation factors of T_H_17 cells that contribute to the inflammatory process. IL-10 expression plays a key role in downregulating immune responses against M.tb bacteria. In the early stages of infection, an increase in IL-6/IL-10 can also increase STAT-3 and SOCS-3, its regulator. This activation of STAT-3/SOCS-3 is related to an immunological response to TB infection. Chronic inflammation after TB infection reduces the function of T cells, especially T_H_17 cells, which explains the low STAT-3/SOCS-3 expression in this study. The other theory that may also explain that low STAT-3/SOCS-3 expression is due to high and sustained type I interferon (IFN) production in the late phase of TB infection (34). High IFN inhibits activation STAT-3 by IL-6 (**Figure 5**). The function of T cells in this study was limited in literature study. However, the study of Kathamuthu in 2022 demonstrated the dominant expansion of CD4, CD8 T, and NK cells expressing Th1/Tc1/Type 1 cytokines in culture-positive LN TB (11) and this may support our hypothetical pathophysiology of tuberculous lymphadenitis.

Until now, there is no observation regarding the CCR-2, CCL-5, IL-6, IL-10, STAT-3, and SOCS-3 in the circulating level (serum or plasma), particularly in tuberculous lymphadenitis. However, tissue-specific macrophages primed with M.tb that shows an elevating level of chemokines CCR-2 and their receptors such as CCL-5 could regulate the movement and interaction of antigen-presenting cells such as DCs and T cells (35) and could further intermediate the recruitment of macrophages and lymphocytes to the infected site (36). Cenicriviroc, an antagonist of CCR-2/CCR-5 (37), might be considered as host-directed therapy for tuberculous lymphadenitis patients along with isoniazid as chemoprophylaxis. However, this needs further study. In addition, Mtb infection induced IL-6 production by macrophages through the activation of STAT3 in lung tubercle tissue as well as the culture supernatant (38). Therefore, the measurement of the circulating level of those cytokines could be another insight in further strengthening the diagnosis and therapy effectivity of TB, particularly in tuberculous lymphadenitis. Furthermore, the expression of the TNF, IFN-α, IFN-β, IL-6, IL-12, and IL-1β should be further investigated to elucidate the detailed immunological mechanism of tuberculous lymphadenitis.

In regard to the current Coronavirus diseases-19 (COVID-19) situation, TB patients might be basically susceptible to COVID-19 infection. T-lymphocyte and macrophage depletion in TB patients play an important role in the severe presentation of COVID-19 (39). The crosslink of inflammatory markers such as IL-6 and IL-10 might contribute to the susceptibility of TB COVID-19 coinfection. Targeting IL-6 and IL-10 might be beneficial to improve the patient’s condition.

## Conclusions

This study shows a preliminary observation of the homing mechanism in tuberculous lymphadenitis mediated by macrophages and lymphocytes. The chronic infection process of tuberculous lymphadenitis was characterized by the expression of IL-10^low^, STAT-3^low^, SOCS-3^low^, CCR-2^high^, CCL-5^high^, and IL-6^high^.

## Data availability statement

The raw data supporting the conclusions of this article will be made available by the authors, without undue reservation.

## Ethics statement

The studies involving human participants were reviewed and approved by Widya Mandala Surabaya’s Health Research Ethics Committee Ref no: 143/WM12/KEPK/DOSEN/T/2021. The patients/participants provided their written informed consent to participate in this study.

## Author contributions

Conceptualization: BN and IT; Methodology: BN, YT, and SW; Formal analysis and investigation: FE, SH, BH, and DJ; Writing—original draft preparation: BN; Writing—review and editing: BN, IT, YT, and SW; Funding acquisition: BN and PT; Resources: IT; Supervision: PT. All authors contributed to the article and approved the submitted version.

## Funding

This study was funded by the Ministry of Research and Technology of the Republic of Indonesia (Ristekdikti/BRIN) ID no. 002/AMD-SP2H/LT-MULTIPDPK/LL7/2021.

## Acknowledgments

We would like to thank St. Vincentius A Paulo Surabaya Hospital for supporting this study and Research Institute Widya Mandala Surabaya Catholic University for the funding assisted.

## Conflict of interest

The authors declare that the research was conducted in the absence of any commercial or financial relationships that could be construed as a potential conflict of interest.

## Publisher’s note

All claims expressed in this article are solely those of the authors and do not necessarily represent those of their affiliated organizations, or those of the publisher, the editors and the reviewers. Any product that may be evaluated in this article, or claim that may be made by its manufacturer, is not guaranteed or endorsed by the publisher.

## Abbreviations

AFB, acid-fast bacilli; CCR-2, C-C chemokine receptor; CCL-5, C-C chemokine ligand; IL, interleukin; M.tb, *Mycobacterium tuberculosis;* STAT-3, signal transducer and activator of transcription 3; SOCS-3, suppressor of cytokine signaling 3; TB, tuberculosis; IHC, immunohistochemistry.
